# Nanosized silver (II) pyridoxine complex to cause greater inflammatory response and less cytotoxicity to RAW264.7 macrophage cells

**DOI:** 10.1186/s11671-015-0848-9

**Published:** 2015-03-19

**Authors:** Avijit Paul, Hee Ju, Sabarinathan Rangasamy, Yumi Shim, Joon Myong Song

**Affiliations:** College of Pharmacy, Seoul National University, 1 Gwanak-ro, Gwanak-ku, Seoul, 151-742 Korea

**Keywords:** Silver pyridoxine nanoparticles, Silver nanoparticles, Macrophage, ROS, IL-8

## Abstract

With advancements in nanotechnology, silver has been engineered into a nanometre size and has attracted great research interest for use in the treatment of wounds. Silver nanoparticles (AgNPs) have emerged as a potential alternative to conventional antibiotics because of their potential antimicrobial property. However, AgNPs also induce cytotoxicity, generate reactive oxygen species (ROS), and cause mitochondrial damage to human cells. Pyridoxine possesses antioxidant and cell proliferation activity. Therefore, in the present investigation, a nanosilver-pyridoxine complex (AgPyNP) was synthesized, and its cytotoxicity and immune response was compared with AgNPs in macrophage RAW264.7 cells. Results revealed that AgPyNPs showed less cytotoxicity compared with AgNPs by producing a smaller amount of ROS in RAW264.7 cells. Surprisingly, however, AgPyNPs caused macrophage RAW264.7 cells to secrete a larger amount of interleukin-8 (IL-8) and generate a more active inflammatory response compared to AgNPs. It activated TNF-α, NF-κB p65, and NF-κB p50 to generate a more vigorous immune protection that produces a greater amount of IL-8 compared to AgNPs. Overall findings indicate that AgPyNPs exhibited less cytotoxicity and evoked a greater immune response in macrophage RAW264.7 cells. Thus, it can be used as a better wound-healing agent than AgNPs.

**Graphical Abstract:**
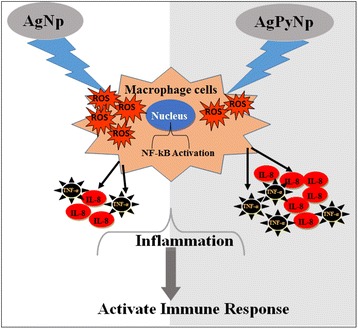
Figurative representation of the comparison of AgNPs and AgPyNPs in macrophage RAW264.7 cells in terms of cytotoxicity and immune response.

Figurative representation of the comparison of AgNPs and AgPyNPs in macrophage RAW264.7 cells in terms of cytotoxicity and immune response.

## Background

Bacterial infections are still a leading cause of death for millions of people worldwide. Infections into wound areas are very common and have caused people immense suffering. Moreover, pathogens have evolved effective approaches to counteract the biocidal action of antibacterial molecules and developed multidrug-resistant strains. As a result, even though many antibiotics have been developed, very few of them have proved effective against multidrug-resistant bacteria. Recently, nanoparticles have been successfully used to deliver therapeutic agents in chronic disease diagnostics, reduce bacterial infections, and act as an antimicrobial agent in the food and clothing industries [1-4]. Silver nanoparticles (AgNPs) have received intensive interest among all of the leading nanotechnology products existing currently, mainly because of their excellent antimicrobial properties and various applications in the medical field [5-7]. Recently, silver nanoparticles have been attracting interest for their clinical applications because of their wound-healing efficacy, which could be exploited in developing better dressings for wounds and ulcers. For topical applications in wound-healing, AgNPs have garnered much attention as antibacterial agents [8-10].

Wound-healing is a complicated procedure involving a combination of activities of different tissues and cell lineages. On injury to the epidermis, a sequence of events is set in motion to repair the wound: the inflammation phase, proliferation phase, and maturation phase. The biochemical mechanisms underlying the wound-healing process involve a number of cytokines and growth factors. These soluble mediators act to regulate chemotaxis, migration, growth, and differentiation of the various cell types present at the injured site. The inflammatory phase starts within minutes after the skin injury, simultaneously with haemostasis. The first inflammatory response is performed by leukocytes, specifically neutrophils, which migrate through the endothelium of the local blood vessels to the wound. The next response is carried out by monocytes, which differentiate into macrophages in the tissues after entering by a mechanism similar to that of neutrophils. These macrophages secrete cytokines and, in this way, initiate an inflammatory response, which results in more cells of the immune system at the place of infection [11]. Tissue macrophages are distributed throughout the whole body and secrete large pools of cytokines when they encounter foreign materials. Cytokines are known as biological-response modifiers that modulate inflammation, immunity, and hematopoiesis. Among them, interleukin-8 (IL-8) is an important cytokine involved in inflammation and postnatal wound-healing by recruiting neutrophils to acute inflammation sites [12]. IL-8 stimulates the chemotaxis of neutrophils and keratinocytes. High concentrations of IL-8 have been observed in burn-wound fluid [13-15]. It has also been reported that silver nanoparticles triggered macrophages to release IL-8 [16]. Despite the rapidly increasing application of AgNPs worldwide, they are highly toxic to various types of cultured cells. It has been reported that AgNP-induced cytotoxicity by producing reactive oxygen species (ROS) and increasing lactate dehydrogenase (LDH) leakage, causing mitochondrial damage and DNA damage [16-19]. Therefore, it is critical to design and develop new therapeutic agents to overcome the silver nanoparticles’ cytotoxicity issues related to wound-healing, as well as antimicrobial activity.

Pyridoxine, a form of vitamin B6, is commonly used as a dietary supplement and therapeutic agent. It plays an important role in maintaining muscle tone in the gastrointestinal tract and promoting the health of the nervous system, skin (preventing dandruff, eczema, and psoriasis), hair, eyes, mouth, and liver. Pyridoxine is essential to normal brain development, participates in the biosynthesis of neurotransmitters, and also controls homocysteine levels in the blood. High levels of homocystein in the blood can be detrimental to the heart muscle. Pyridoxine also possesses antioxidant activity [20] and cell proliferation facilitation properties [21,22].

The main aim of this study was to prepare a nanosilver-pyridoxine complex (AgPyNP) and investigate its ROS-production-based antimicrobial activity, cytotoxicity, and inflammatory action related to its wound-healing ability in the RAW264.7 macrophage cell line. AgNPs were used as a model of comparison for the novel nanoparticle. Discovery of a new nanoparticle that causes more vigorous inflammatory action and less cytotoxicity than AgNPs while its antibacterial activity is maintained as a topical therapeutic agent, will pave the way for the next generation of therapeutic nanoparticles. Macrophage cells were used for this study to assess the inflammatory response of AgPyNPs, as well as AgNPs.

## Methods

Dulbecco’s modified Eagle’s medium (DMEM), fetal bovine serum (FBS), penicillin, streptomycin, neomycin (PSN) antibiotic, trypsin, and ethylenediaminetetraacetic acid (EDTA) were purchased from Gibco BRL (Grand Island, NY, USA). Tissue culture plastic wares were obtained from BD Biosciences (San Jose, CA, USA). Propidium iodide; acrydine orange; 4′,6′-diamidino-2 phenylindole (DAPI); 3-(4,5-Dimethylthiazol-2 yl)-2, S-diphenyltetrazolium bromide (MTT); and all other chemicals used were purchased from Sigma-Aldrich Co. (St. Louis, MO, USA). All organic solvents used were of HPLC grade. Pyridoxine, silver nitrate (AgNO_3_), ammonium hydroxide (NH_4_OH), ammonium persulfate ((NH_4_)_2_S_2_O_8_), dioctyl sulfosuccinate (AOT), dimethyl sulfoxide (DMSO), sodium citrate, sodium borohydride (NaBH_4_), polyvinylpyrrolidone (PVP), and heptane utilized for the synthesis of nanoparticles were also purchased from Sigma-Aldrich Co. (St. Louis, MO, USA).

### Synthesis of AgNPs

**Figure 1:**
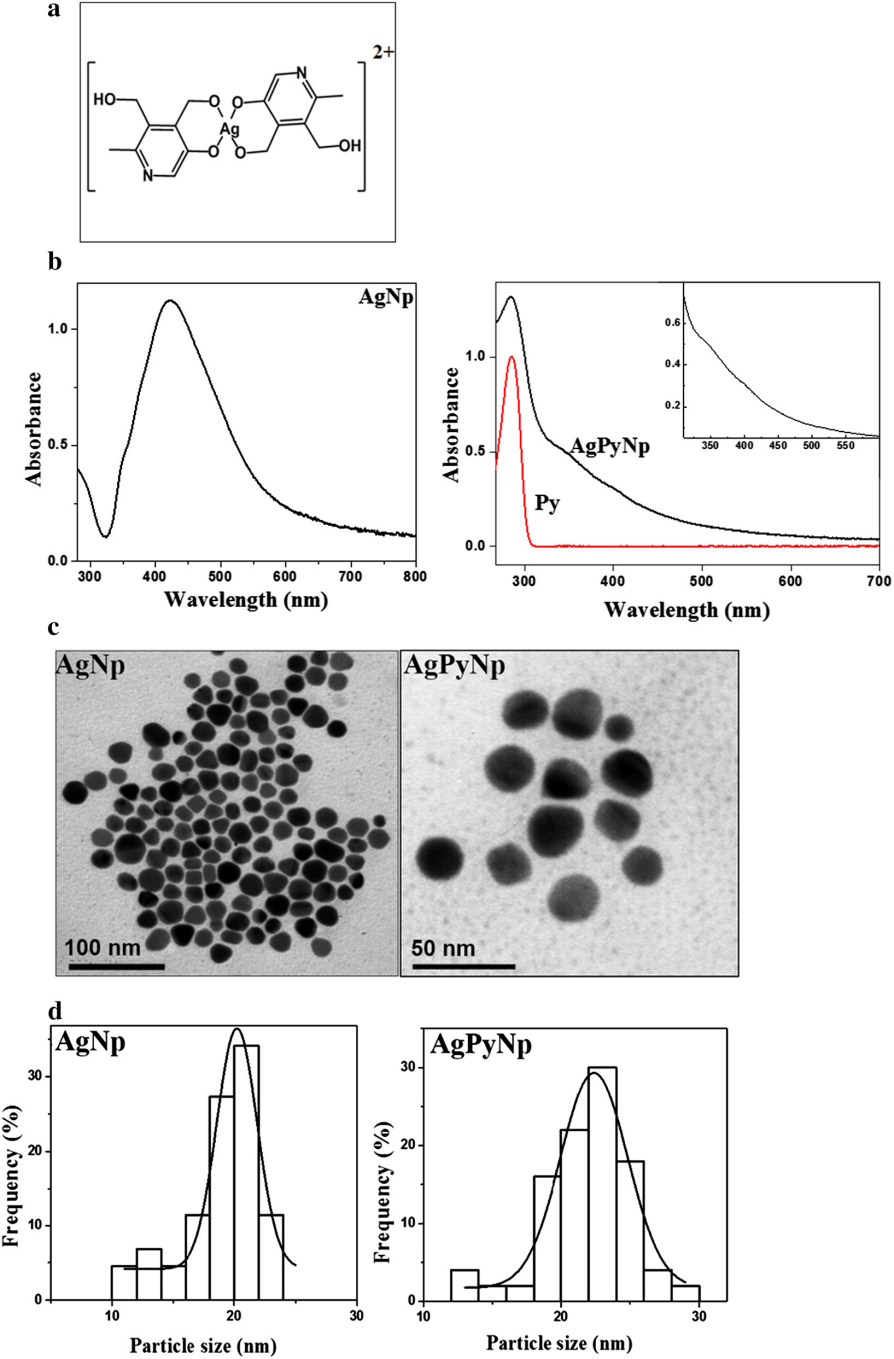
**Chemical structure, absorption spectra, TEM image, and size-distribution analyses. (a)** Chemical structure of nanosized silver (II) pyridoxine complex; **(b)** absorption spectra of AgNPs and AgPyNPs; **(c)** TEM image of AgNPs and AgPyNPs synthesized by a modified citrate-reduction method and reverse microemulsion method, respectively; **(d)** size distribution analysis of synthesized nanoparticles. AgNPs, silver nanoparticles; AgPyNPs, nanosilver-pyridoxine complexes.

Small-sized, highly monodispersed AgNPs were synthesized according to the modified literature procedure by Pham Van Dong et al. by reducing aqueous AgNO_3_ in the presence of NaBH_4_ and PVP as reducing and stabilizing agents. Briefly, an aqueous solution of sodium citrate (0.25 mL, 15 mM) was added to a flask containing 25 mL of deionized water. Then, aqueous solution of AgNO_3_ (0.5 mL, 2.5 mM) was added and gently mixed. Freshly prepared NaBH_4_ (0.25 mL, 25 mM) was rapidly added and allowed to stir for 30 s. Finally, aqueous solution of PVP (0.25 mL, 2.5 mg/mL) was added to the suspension and stirred further for 30 min. Dark yellow coloured suspension was centrifuged and the pellet was dried under vacuum. Absorption spectroscopy is primarily used for the structural characterization of AgNPs. From the absorption spectrum, the surface plasmon resonance peaks of the silver nanoparticles were observed around 420 nm, confirming the formation of spherical silver nanoparticles (Figure 1b). The size and morphology of nanoparticles were analysed by transmission electron microscope (TEM). The TEM images confirmed the monodispersity of nanoparticles (Figure 1c). The size-distribution analysis was used to determine the average particle size at 20 nm (Figure 1d).

### Synthesis of AgPyNPs

AgPyNPs were synthesized by a modified reverse microemulsion (W/O) technique. To perform a complexation reaction under the condition of microemulsion, AgNO_3_, pyridoxine, and ammonium persulfate were taken at a 1:4:4 mole ratio. W/O microemulsion was initiated by dissolving AOT (0.44 g, 0.1 M) surfactant in 10 mL heptane. Then, pyridoxine (3.9 mg) was dissolved in 116 μL of a 1:1 DMSO/water mixture. Aqueous AgNO_3_ (29 μL) was added to the heptane solution to form a stable microemulsion. Ammonium hydroxide (5.8 μL, 29 wt.%) was added to the reaction mixture and gently stirred for 30 min. The microemulsion solution turned brown, indicating the oxidation of silver to a higher valence state. The microemulsion solution was allowed to stir for another 24 h. AgPyNPs were isolated by disintegrating the microemulsion solution using ethanol or acetone. The nanoparticles were recovered by centrifugation. Finally, nanoparticles were washed twice with methanol to remove the unreacted starting materials, surfactants, and organic solvent. Then, nanoparticles were dried under vacuum and stored as a powder under room temperature. Figure 1b shows the electronic absorption spectrum of AgPyNPs. The nanosized complex formation between silver and pyridoxine ligand (Py) was confirmed through the analysis of the spectrum. AgPyNPs exhibit different absorption patterns compared to that of Py. AgPyNPs showed strong absorption in the wavelength region larger than 300 nm. The characteristic peaks at 345 and 400 nm were also present as shoulders. Nanoparticle formation was further confirmed by TEM. Figure 1c shows the monodispersed AgPyNPs and their average particle size was determined to be 20 nm (Figure 1d).

*1H NMR* (500 MHz, DMSO-d6) 7.86 (s, 2H), 4.71 (s, 4H), 4.47 (s, 4H), 2.39 (s, 6H); *IR* (KBr, cm^−1^) 3432.67 (s, *v*_OH_), 1631.48 (w, *v*_CO_^−^), 1403.92 (m, *v*_C-C_); *EA* (C, H, N) Anal. Calcd for C_16_H_20_N_2_O_7_Ag: Ag, 23.44; C, 41.76; H, 4.38; N, 6.09; O, 24.34. Found: Ag, 23.35; C, 41.71; H, 4. 32; N, 6.15; O, 24.39. *FAB-MS* (*m/z*): 460.21.

### Cell culture

RAW264.7 cells (KCLB®, Seoul, South Korea) were cultured in standard (DMEM) medium (Gibco, Grand Island, NY, USA) supplemented with heat-inactivated 10% FBS (Gibco, Grand Island, NY, USA), penicillin (60 μg/mL), and streptomycin (100 μg/mL) solutions in cell-culture flasks (75 cm^2^). Cells were grown at 37°C in a humidified incubator containing 5% CO2 (US AutoFlowt, NuAire, Plymouth, MN, USA). After the cells attained confluence, they were subcultured following trypsinization with a 0.25% trypsin-EDTA solution (Gibco, Grand Island, NY, USA).

### MTT assay

To determine the effect of AgNPs and AgPyNPs on RAW264.7 cell growth, cultured cells were treated with different doses of AgNPs and AgPyNPs ranging up to 70 μg/mL. After 24 h of treatment, cell viability was assessed using MTT assay. The intracellular formazan crystals formed were solubilized with acidic isopropanol, and the absorbance of the solution was measured at 595 nm. Percentage viability was calculated as follows: (OD of drug treated sample/OD of control sample) × 100.

### Measurement of intracellular ROS

Estimation of intracellular ROS was performed using cell-permeable fluorescent-probe 2′-7′-dichlorofluorescein-diacetate (DCFDA), a non-fluorescent compound, which is converted into highly fluorescent dichlorofluorescein (DCF) by cellular peroxides. Cells, after being treated with AgNPs/AgPyNPs for the desired amount of time, were loaded with 10 μM DCFDA. Following the incubation period at 37°C for 30 min in the dark, cells were washed with PBS and fluorescence-monitored with a fluorescence microscope (Axisocope Plus 2, Zeiss, Jena, Germany). The level of intracellular ROS was also measured by FACS with an excitation wavelength of 480 nm and an emission wavelength of 530 nm using the same fluorescence probe.

### Analysis of IL-8 release

The expression level of IL-8 secretion of AgNP- and AgPyNP-treated RAW264.7 cells were analysed by the ELISA method using an IL-8 assay kit (R&D systems, Minneapolis, USA) according to the manufacturer’s protocol.

### Effect of AgNPs and AgPyNPs on TNF-α, NF-κB p65, and NF-κB p50 expression

AgNP/AgPyNP-induced expressions of TNF-α, NF-κB p65, and NF-κB p50 in RAW264.7 cells were monitored using quantitative cellular imaging cytometry. TNF-α, NF-κB p65, and NF-κB p50 antibodies were purchased from BD Biosciences (San Jose, CA, USA) and followed by conjugate with quantum-dot according to the manufacturer’s protocol (Life Science Technology, Grand Island, NY, USA). After AgNP/AgPyNP treatment, cells were washed with PBS and incubated with quantum-dot-conjugated TNF-α, NF-κB p65, and NF-κB p50 antibodies according to the protocol. The emission maxima for TNF-α, NF-κB p65, and NF-κB p50 at 525, 565, and 625 nm, respectively, were imaged using an AOTF microscope.

## Results

### Cell viability assay

**Figure 2:**
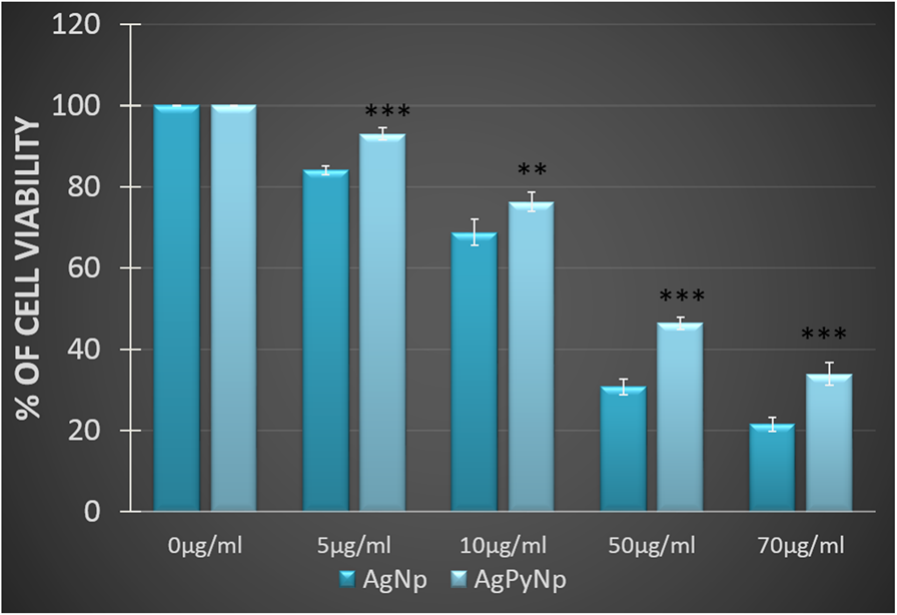
**AgNP- and AgPyNP-induced cytotoxicity in RAW264.7 cells was analysed using MTT assay.** Cells were treated with different concentrations of AgNP and AgPyNP for 24 h. The values represent the mean of at least three independent experiments normalized to untreated controls. Error bars represent the standard deviations of three independent experiments. Student’s *t*-test was used to determine the statistical significance: **P < 0.01; ***P < 0.001. Presented data were combined from at least three experiments. AgNPs, silver nanoparticles; AgPyNPs, nanosilver-pyridoxine complexes.

Cell viability assay was performed by MTT assay using different dosages of AgNPs/AgPyNPs in RAW264.7cells after 24 h of treatment. Untreated cells served as controls. The results of the MTT assay are shown in Figure 2. Both AgNPs/AgPyNPs showed their cytotoxicity at concentrations of 50 μg/mL and higher. AgNPs exhibited greater cytotoxicity compared to that of AgPyNPs. The 84.1%, 68.78%, 30.79%, and 21.56% viable cells were observed after 5, 10, 50, and 70 μg/mL AgNP treatments, respectively. Whereas, 92.96%, 76.28%, 46.47%, and 33.99% viable cells were observed after 5, 10, 50, and 70 μg/mL AgPyNP treatments, respectively, for 24 h. This result indicated that AgPyNPs was less cytotoxic compared to AgNPs.

### Effect of AgNPs and AgPyNPs on ROS generation

**Figure 3:**
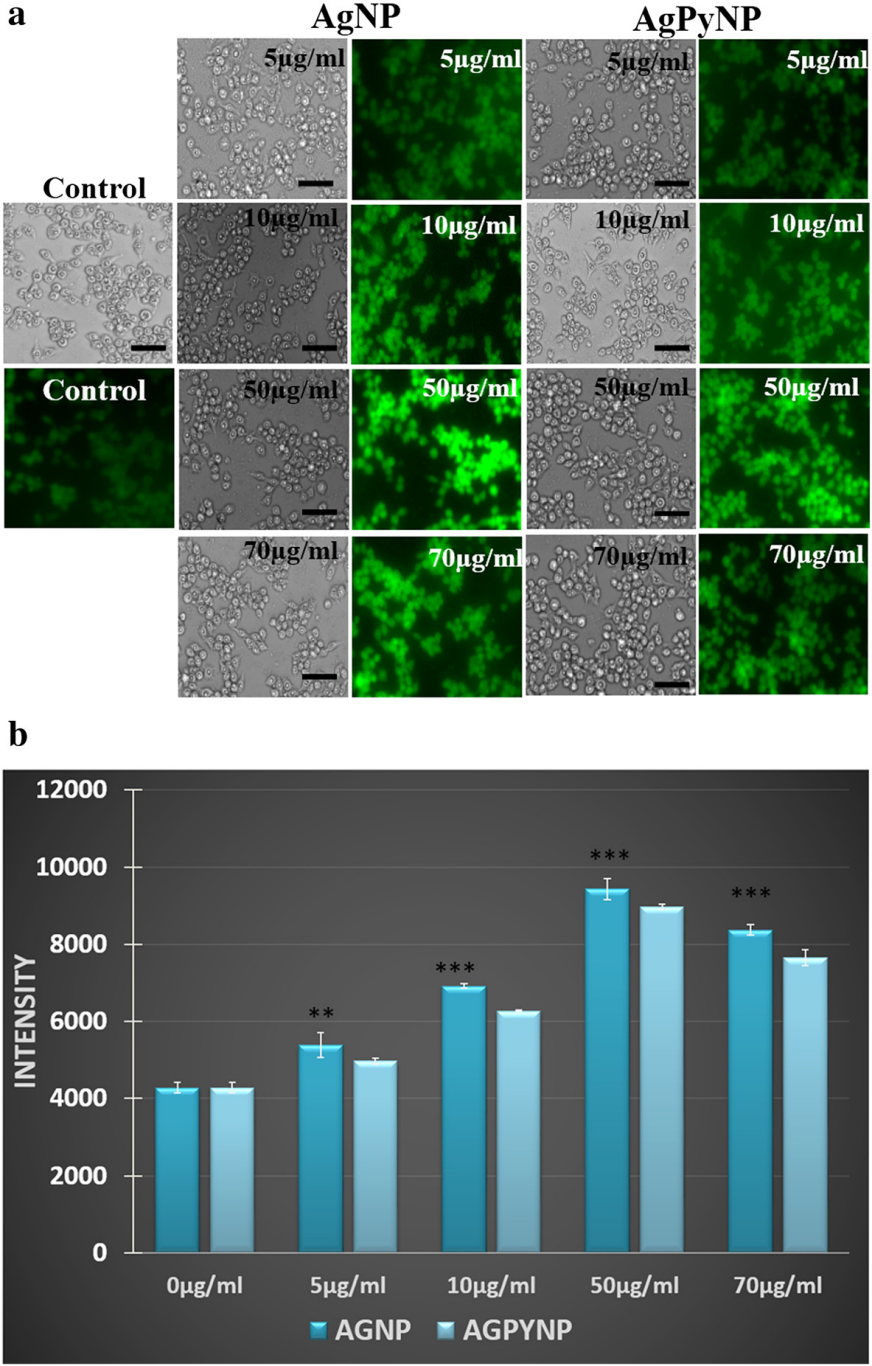
**Fluorescence and bar graph of AgNP- and AgPyNP-treated RAW264.7 cells. (a)** Fluorescence microscopic analysis of ROS generation in AgNP- and AgPyNP-treated RAW264.7 cells and scale bar represents 35 μm. **(b)** Bar graphs represent the mean ± SD intensity values of triplicate treatment groups to analyse the ROS generation in AgNP- and AgPyNP-treated RAW264.7 cells. Error bars represent the standard deviations of three independent experiments. Student’s *t*-test was used to determine the statistical significance: **P < 0.01; ***P < 0.001. Presented data were combined from at least three experiments. AgNPs, silver nanoparticles; AgPyNPs, nanosilver-pyridoxine complexes.

**Figure 4:**
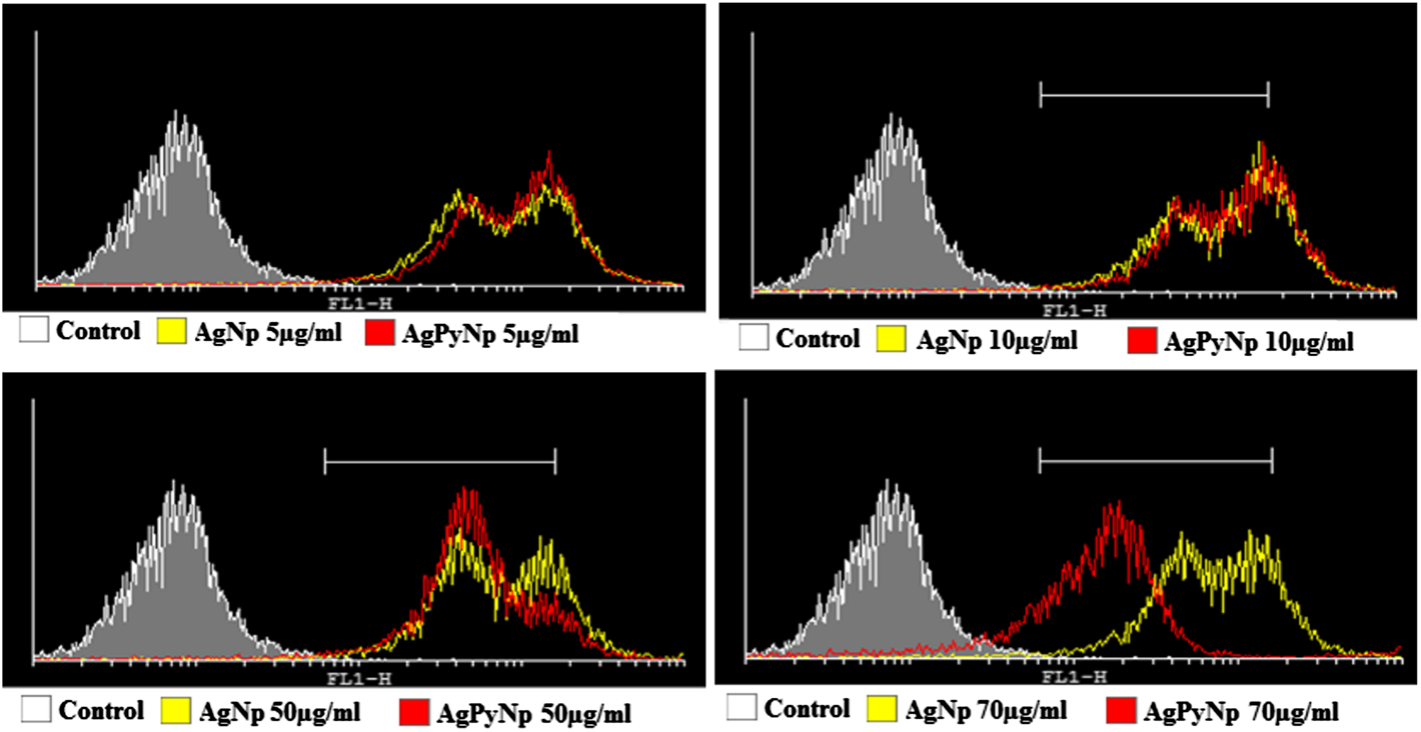
**Flow-cytometric analysis of ROS generation in AgNP- and AgPyNP-treated RAW264.7 cells.** Peak shifts towards the right indicate higher fluorescence intensities, which signify higher ROS production. Whereas, peak shifts towards the left indicate lower fluorescence intensities, which signify lower ROS production. AgNPs, silver nanoparticles; AgPyNPs, nanosilver-pyridoxine complexes.

The mechanism underlying the induction of inflammation by AgNPs and AgPyNPs may be through the generation of ROS. Therefore, the production of ROS was measured by using fluorescence microscopy as well as flow cytometry. Both the fluorescence microscopic image (Figure 3a,b) and flow-cytometric data (Figure 4) suggest that both AgNPs and AgPyNPs generate ROS in a concentration-dependent manner, in which ROS generation increases with the increasing concentrations of AgNP and AgPyNP. However, AgNPs produce more ROS than AgPyNPs. Due to high cytotoxicity, the levels of ROS production were reduced at high concentrations of AgNPs and AgPyNPs (70 μg/mL). From the flow-cytometric observation, it was clear for both 5 and 10 μg/mL of AgNPs and AgPyNPs that almost the same amount of ROS was produced. Whereas, the peak of ROS produced by 50 μg/mL AgPyNP-treated cells (Figure 4) was shifted towards the left compared to 50 μg/mL AgNP-treated cells. After treatment of 70 μg/mL AgPyNPs, the peak of ROS was shifted more towards the left as compared to 70 μg/mL AgNP-treated cells. This result signifies that, due to the antioxidant activity of pyridoxine, the synthesized silver pyridoxine nanoparticles scavenge the ROS; thus, a smaller amount of ROS production was observed in the case of AgPyNPs compared to AgNPs.

### Assessment of IL-8 secretion

**Figure 5:**
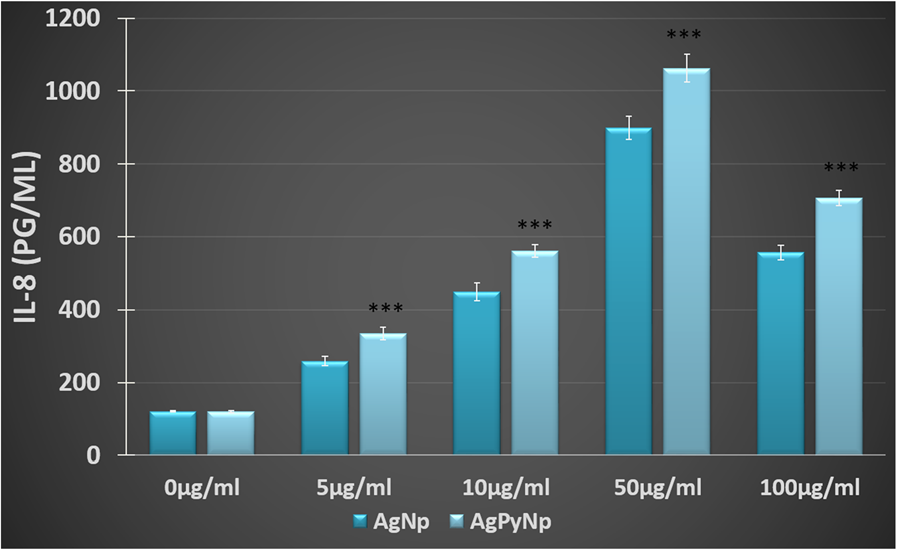
**IL-8 secretion in cell culture supernatants were assessed by ELISA after AgNP and AgPyNP treatment in RAW264.7 cells.** The values represent the mean of at least three independent experiments normalized to untreated controls. Error bars represent the standard deviations of three independent experiments. AgNPs, silver nanoparticles; AgPyNPs, nanosilver-pyridoxine complexes; IL-8, interleukin-8.

To assess IL-8 concentration in cell culture supernatants, ELISA was performed. As shown in Figure 5, IL-8 secretion was concentration-dependent. With increasing concentrations of AgNPs and AgPyNPs, IL-8 secretion also increased and reached a maximum at 50 μg/mL. At 50 μg/mL, IL-8 secretion was reduced due to toxicity mediated by high concentrations of AgNPs and AgPyNPs. In this case, AgPyNPs secreted more IL-8 than AgNPs. From Figure 5, it was clearly observed that, up to 50 μg/mL, the IL-8 secretion was continuously increased in a concentration-dependent manner for both AgNP- and AgPyNP-treated cells; at 50 μg/mL, it decreased with increases in concentration of AgNPs, as well as AgPyNPs. This decrease in IL-8 secretion was due to the cytotoxicity of higher concentrations of both AgNPs and AgPyNPs. However, for all of the concentrations of AgPyNP treatment, the IL-8 secretion was higher when compared with AgNP-treated cells. These results indicated that AgPyNPs enhanced the immune protection ability of macrophage cells by secreting more IL-8 compared to AgNPs.

### Assessment of TNF-α, NF-κB p65, and NF-κB p50 expression

**Figure 6:**
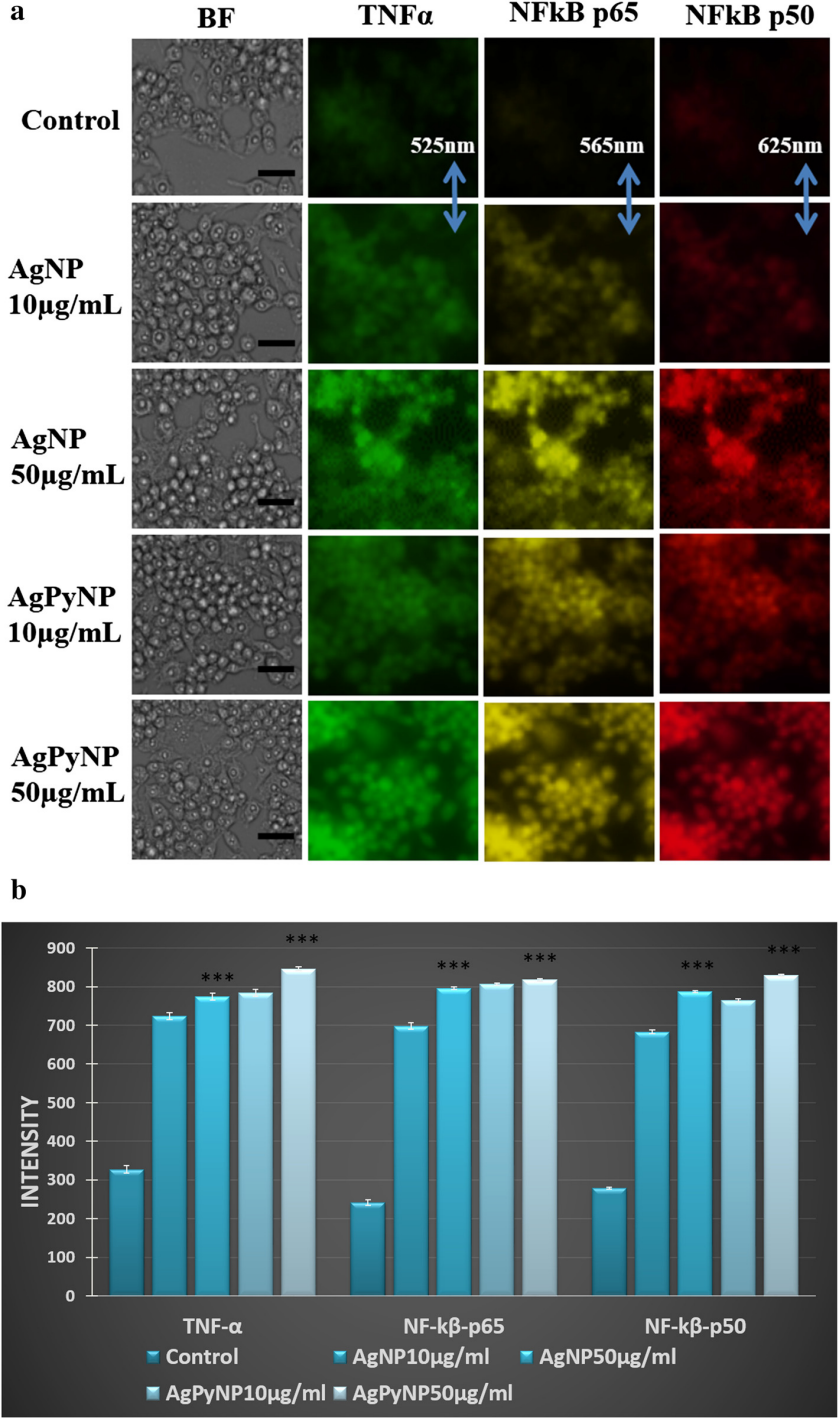
**Fluorescent images and bar graphs of AgNP- and AgPyNP-treated RAW264.7 cells. (a)** Photographs represent TNF-α, NF-κB p65, and NF-κB p50 activation after AgNp and AgPyNp treatment in RAW264.7 cells, and the scale bar represents 15 μm. Fluorescent images were taken at 525 nm (TNF-α), 565 nm (NF-κB p65), or 625 nm (NF-κB p50). **(b)** Bar graphs represent the mean ± SD intensity values of triplicate treatment groups to analyse the ROS generation in AgNP- and AgPyNP-treated RAW264.7 cells. Error bars represent the mean ± SD of three independent experiments. Student’s *t*-test was used to determine the statistical significance: ***P < 0.001. Presented data were combined from at least three experiments. AgNPs, silver nanoparticles; AgPyNPs, nanosilver-pyridoxine complexes; BF, bright field.

High-content cellular imaging cytometry was used to analyse the expressions of TNF-α, NF-κB p65, and NF-κB p50. As shown in Figure 6a,b, the expression of TNF-α, NF-κB p65, and NF-κB p50 increased with AgNP and AgPyNP treatments compared to that of untreated cells. Here, AgPyNPs exhibited a greater activation of TNF-α, NF-κB p65, and NF-κB p50 than AgNPs. From Figure 6a,b, it was clearly observed that, for all of the cases, the AgPyNP-treated cells phosphorylated more TNF-α, NF-κB p65, and NF-κB p50 than AgNP-treated cells. These results indicated that AgPyNPs strengthened the inflammation response by activating TNF-α and NF-κB and, thus, contributed to the enhancement of the wound-healing process better than AgNPs.

## Discussion

Nanotechnology is growing in importance for improving the quality of human life through its impact on medicine and drug delivery systems. The antiseptic action of silver has been well known for centuries. As a result, this precious metal has long been used to treat infectious diseases and heal wounds. Over the last decade, silver-nanoparticle-mediated therapeutic research attracted the intense interest of many researchers.

AgNPs promote wound-healing through their powerful antibacterial properties [23,24]. They induce inflammatory response on macrophage cells by releasing IL-8 [16]. Nanosilver particles and released ions from oxidized nanosilver surfaces readily bind to sulfur- and phosphorus-containing biomolecules, such as proteins and DNA, thereby potentially causing cell damage [25-28]. This observed toxicity is also related to free radical (ROS) formation that can damage the cell membrane [29]. Upon internalization of nanosilver by cells, Ag metal could be further dissolved to Ag^+^ ions in the lysosomes due to the lower pH found there. Such Ag^+^ ions could also interfere with mitochondrial activity and induce cytotoxicity [30]. Therefore, in the present research, we prepared nanoparticles consisting of a silver-pyridoxine complex to reduce the undesired cytotoxicity of silver nanoparticles, as pyridoxine possesses antioxidant activity [20] and also enhances cell proliferation [21,22,31]. Observing the considerable antimicrobial effects of AgPyNPs in our laboratory (data not shown) lead to an increase in interest in studying the cytotoxicity levels of AgPyNPs. We compared the cytotoxicity and immune response of AgNPs and AgPyNPs with respect to IL-8 secretion on the macrophage RAW264.7 cell line. In our study, AgPyNPs showed less cytotoxicity on the macrophage cell line RAW264.7 as compared to AgNPs, which is a beneficial feature in producing primary defence by macrophages for combating bacteria. Therefore, we further analysed the ROS-production ability of AgPyNPs. From our fluorescence microscopic investigation and flow-cytometric analysis, it was clear that the ROS-production ability of AgPyNPs in RAW264.7 cells was slightly inferior compared to AgNPs. This inferior ROS production is due to the antioxidative property of pyridoxine, which scavenges the ROS produced by silver ions [32]. These phenomena motivate our in-depth study to analyse the immune effect of AgPyNPs. Macrophages represent a primary line of defence against foreign materials. The macrophage phenotype generates ROS, as well as a plethora of inflammatory cytokines that facilitate killing of invading pathogens [33]. Tissue macrophages are distributed throughout the whole body and secrete large pools of cytokines when they encounter foreign materials or pathogens. Cytokines are inflammatory mediators produced during an immunological response. An increase in the release of cytokines indicates that an external stimulation of immune cells is occurring. Among the cytokines participating in innate immunity, IL-8 recruits neutrophils to acute inflammation sites. It is reported that silver nanoparticles induce IL-8 secretion [16,34]. In this current study, we found that AgPyNPs induce more IL-8 secretion in RAW264.7 cells compared to AgNPs. This result suggests that AgPyNPs have more ability to produce primary antimicrobial immune defence than AgNPs. Compared to AgNPs, the synthesized AgPyNPs showed less cytotoxicity and also enhanced IL-8 in RAW264.7 cells. These phenomena indicated that, due to the antioxidant and cell proliferation activity of pyridoxine, the synthesized AgPyNPs provided extra capability to macrophage cells to further enhance the immune response to fight microbial infection by secreting IL-8, when compared with AgNPs.

Pro-inflammatory genes, such as TNF-α and transcription factor NF-κB, have been reported to induce the expression of the genes associated with immunological and cellular defence systems [35,36]. There have also been some reports that suggest that carbon black nanoparticles and silica nanoparticles have the ability to induce inflammatory response by activation of NF-κB in macrophages [37]. Therefore, in our present study, we analysed the expressions of TNF-α and NF-κB as an upstream event of nanoparticle-induced inflammatory response in macrophage cells using a high-content cellular imaging cytometry method. Our screening method, based on cellular imaging cytometry, provides simultaneous monitoring of cellular events and facilitates quantitative multivariate cellular analysis with the elimination of false positive errors, as it provides high-spectral resolution among the fluorophores. Our results revealed that expressions of TNF-α, NF-κB p65, and NF-κB p50 were increased with treatment of both AgNPs, as well as AgPyNPs, compared to control. These results demonstrated that AgNPs/AgPyNPs significantly increase the translocation of p65 and p50 subunits of NF-κB to nucleus. Thus, inflammatory response in RAW264.7 cells is induced and facilitates the release of IL-8. Then, we compared the expressions of TNF-α, NF-κB p65, and NF-κB p50 after AgNP and AgPyNP treatment in macrophage cells. Here, we also found that AgPyNPs induced TNF-α, NF-κB p65, and NF-κB p50 expressions better than AgNPs. As revealed in earlier studies [22,31], pyridoxine helps to enhance the cellular proliferation of macrophage cells, which leads to an increased immune response. Their studies revealed the enhanced inhibition of microbial infection as a result of strengthened immune response by pyridoxine. In accordance with the previous studies, it is thought that AgPyNPs possessing pyridoxine domain showed greater immune response to combat microbial infections by activation of NF-κB, which helps to release more IL-8 and TNF-α as compared to AgNPs.

## Conclusions

Overall results demonstrate that silver pyridoxine nanoparticles exhibit a better immunological response with low cytotoxicity compared to silver nanoparticles, which is a powerfully beneficial feature to heal wounds and infection-related issues. Due to its antioxidant property, silver pyridoxine nanoparticles generate a lesser amount of ROS that produce oxidative stress in phagocytic cells, such as macrophage. Thus, silver pyridoxine nanoparticles help macrophages secrete more IL-8, which is implicated in inflammation and postnatal wound-healing. IL-8 leads to increased reepithelialization of wounds by stimulating keratinocyte proliferation and migration [38]. Therefore, it can be concluded that the newly formulated silver pyridoxine nanoparticles exhibit better therapeutic ability compared to silver nanoparticles for treating and healing wounds.

## Abbreviations

AgNPs: silver nanoparticles
AgPyNP: nanosilver-pyridoxine complex
DMEM: Dulbecco’s modified Eagle’s medium
EDTA: ethylenediaminetetraacetic acid
FBS: fetal bovine serum
IL-8: interleukin-8
LDH: lactate dehydrogenase
